# Sex-Differences in Renal Expression of Selected Transporters and Transcription Factors in Lean and Obese Zucker Spontaneously Hypertensive Fatty Rats

**DOI:** 10.1155/2015/483238

**Published:** 2015-01-29

**Authors:** Andrea Babelova, Birgitta C. Burckhardt, Waja Wegner, Gerhard Burckhardt, Maja Henjakovic

**Affiliations:** ^1^Institute for Cardiovascular Physiology (Physiology I), Faculty of Medicine, Goethe-University, Theodor-Stern-Kai 7, 60596 Frankfurt am Main, Germany; ^2^Cancer Research Institute, Slovak Academy of Sciences, Vlarska 7, 83391 Bratislava, Slovakia; ^3^Institute for Systemic Physiology and Pathophysiology, University Medical Center Göttingen, Humboldtallee 23, 37073 Göttingen, Germany

## Abstract

The aim of this study was to identify sex-dependent expression of renal transporter mRNA in lean and obese Zucker spontaneously hypertensive fatty (ZSF1) rats and to investigate the interaction of the most altered transporter, organic anion transporter 2 (Oat2), with diabetes-relevant metabolites and drugs. Higher incidence of glomerulosclerosis, tubulointerstitial fibrosis, and protein casts in Bowman's space and tubular lumen was detected by PAS staining in obese male compared to female ZSF1 rats. Real-time PCR on RNA isolated from kidney cortex revealed that Sglt1-2, Oat1-3, and Oct1 were higher expressed in kidneys of lean females. Oct2 and Mrp2 were higher expressed in obese males. Renal mRNA levels of transporters were reduced with diabetic nephropathy in females and the expression of transcription factors Hnf1*β* and Hnf4*α* in both sexes. The highest difference between lean and obese ZSF1 rats was found for Oat2. Therefore, we have tested the interaction of human OAT2 with various substances using tritium-labeled cGMP. Human OAT2 showed no interaction with diabetes-related metabolites, diabetic drugs, and ACE-inhibitors. However, OAT2-dependent uptake of cGMP was inhibited by furosemide. The strongly decreased expression of Oat2 and other transporters in female diabetic ZSF1 rats could possibly impair renal drug excretion, for example, of furosemide.

## 1. Introduction

Diabetes mellitus is one of the most common diseases, with 346 million affected individuals worldwide in 2012 and represents the seventh leading cause of death in the United States [1]. Type 2 diabetes accounts for about 90% of all diagnosed cases [2]. More than twenty percent of patients with type 2 diabetes develop diabetic nephropathy [3]. Moreover, clinical studies reported a high prevalence of hypertension for patients in both early and late stages of this disease, which potentiates further progression of kidney damage [4]. Vice versa, the decline in kidney function contributes to elevated blood pressure in patients with type 2 diabetes [4]. Premenopausal women typically have lower blood pressure than age-matched men, possibly mediated by estradiol which appears to act as a vasodilator [5]. This is in line with a higher incidence of diabetic nephropathy associated with type 2 diabetes observed in men compared to age-matched women [6].

An unresolved issue is the association of diabetic nephropathy with expression of transport proteins responsible for renal secretion of drugs. Members of the solute carrier 22 (Slc 22) gene family, organic anion transporters (human OAT; rat and mouse Oat), and organic cation transporters (human OCT; rat and mouse Oct) are expressed in the kidneys and take up endogenous and exogenous compounds, including frequently prescribed drugs, from the blood into proximal tubular cells [7–10]. Among antidiabetic drugs, OCT2 is involved in proximal tubular secretion of metformin, and OAT3 transports sitagliptin [9, 11]. For rat kidneys, androgen-dependent expression of Oat1, Oat3, and Oct2 and higher expression of Oat2 in females was reported, suggesting sex-dependent renal drug handling at least in this species [12, 13].

ATP-dependent efflux transporters, multidrug resistance-associated protein 2 (Mrp2), Mrp4, and P-glycoprotein (Mdr1b) are localized in the apical membrane of renal proximal tubules and are responsible for the secretion of organic anions and cations from the proximal tubular cells into the urine [14]. Human gene promoters of OAT1, OAT2, and MRP2 are activated by the transcription factor hepatocyte nuclear factor 4*α* (HNF4*α*) [15–17]. Interestingly, single nucleotide polymorphisms (SNPs) associated with type 2 diabetes were found in the gene encoding HNF4*α* [18].

The aim of this study was to identify, at the level of mRNA, potential sex- and diabetes-dependent changes of Oats, Octs, ATP-dependent efflux transporters, and the transcriptional regulators, Hnf1*α*, Hnf1*β*, and Hnf4*α*. Additionally, the levels of sodium-dependent glucose cotransporter 1 (Sglt1) and Sglt2 were investigated. We used obese Zucker spontaneously hypertensive fatty rats (ZSF1) as an established animal model for type 2 diabetes and diabetic nephropathy. Lean ZSF1 rats served as a model-specific control.

ZSF1 rats were previously developed by crossing rat strains with different mutations in the leptin receptor gene, Zucker diabetic fatty (ZDF) rats, and spontaneously hypertensive heart failure (SHHF) rats [19, 20]. Lean and obese ZSF1 rats had similar mean arterial blood pressure at seven weeks of age and elevated blood pressure (BP) at 20 weeks of age (Table 1) [21, 22]. The concentration of blood glucose and plasma triglycerides were higher in obese than in lean ZSF1 rats at eight weeks of age (Table 1) [19]. However, only obese ZSF1 rats developed type 2 diabetes with diabetic nephropathy, characterized by elevated urine albumin/creatinine ratios (Table 1) [19, 23]. Our hypothesis was that, in addition to sex-dependences, the renal expression of Oats and Octs may be altered in diabetic nephropathy that might influence the renal secretion of metabolites and exogenous substances.

## 2. Material and Methods

### 2.1. Animals and Kidney Preservation

Kidneys from obese male and female ZSF1 (ZSF1-*Lepr* ^*fa*^ *Lepr* ^*cp*^/Crl) rats, and their lean controls, were obtained from Charles River (Stone Ridge, NY). Animals were kept in the animal facility of Charles River Laboratories under conventional housing conditions (22°C, 55% humidity, and 12 h day/night cycle) with free access to water and rat chow. Kidneys of adult (16-week-old) ZSF1 rats were removed postmortem in accordance to federal law, conserved in paraformaldehyde (4%) or RNA*later*, and shipped to our laboratory.

### 2.2. Morphological Study

Rat kidneys fixed in paraformaldehyde (4%) were processed for embedding in paraffin. Serial sections (3 *μ*m) of kidneys were stained with periodic acid-Schiff (PAS).

### 2.3. RNA Isolation

After removal of the kidney capsule, cortical slices were prepared, from which total RNA was isolated using RNeasy Mini Kit (QIAGEN, Hilden, Germany) according to the manufacturer's recommendations. Quality and quantity of the extracted RNA were determined using the Agilent 2100 Bioanalyzer (Agilent Technologies, Boeblingen, Germany) and NanoDrop ND-1000 Spectrophotometer (Thermo Scientific NanoDrop Technologies, Wilmington, NC), following the manufacturer's protocol. RNAs with RNA integrity number (RIN) > 8 were used for further experiments.

### 2.4. cDNA Synthesis and TaqMan Real-Time PCR

Superscript II Reverse Transcriptase (Life Technologies, Darmstadt, Germany) and Oligo dT-Primers (Eurofin MWG Operon, Ebersberg, Germany) were used for reverse transcription of RNA. Genes of interest were analyzed using TaqMan Master Mix and TaqMan Gene Expression Assays (Life Technologies): Sodium-dependent glucose cotransporter 1 (Sglt1), Rn00564718_m1; Sglt2, Rn00574917_m1; Oat1, Rn00568143_m1; Oat2, Rn00585513_m1; Oat3, Rn00580082_m1; Oct1, Rn00562250_m1; Oct2, Rn00580893_m1; Mrp2, Rn00563231_m1; Mrp4, Rn01465702_m1; P-glycoprotein (Mdr1b), Rn00561753_m1; Hnf1*α*, Rn00562020_m1; Hnf1*β*, Rn00447453_m1; Hnf4*α*, Rn00573309_m1. The mRNA levels of hypoxanthine phosphoribosyltransferase 1 (Hprt1, Rn01527840_m1), *β*-actin (Rn00667869_m1), and cyclophilin A (Rn00690933_m1) were tested as housekeeping control genes for sample normalization. For all tested genes, PCR conditions were as follows: 2 min at 50°C followed by 10 min at 95°C and 40 amplification cycles (95°C for 15 s and 60°C for 60 s), using Mx3000P real-time PCR cycler (Agilent Technologies). Signals detected between 35–40 amplification cycles were defined as low gene expression. The amplification efficiencies of used assays were 100% (+/− 10%), in accordance with manufacturer's information. The real-time PCR data were analyzed as ΔCt = housekeeping gene (Hprt1)-gene of interest (Figure 2), using the 2^−ΔΔCt^ method (see Supplementary Figures in Supplementary Material available online at http://dx.doi.org/10.1155/2015/483238) [25].

### 2.5. Transport Studies

The uptake of cGMP in HEK293 cells stably transfected with human OAT2 (kindly provided by PortaCellTec Biosciences GmbH, Göttingen, Germany) was investigated in the absence and presence of metabolites known to be accumulated in diabetic patients and therapeutics for treatment of diabetes and hypertension. First, OAT2- and vector-transfected HEK293 cells were seeded at a density of 2 · 10^5^ cells/well in a 24-well cell culture plate and incubated for ~72 h in Dulbecco's modified Eagle medium-high glucose (DMEM HG, D5796, Sigma Aldrich) culture medium supplemented with 10% fetal bovine serum (number 10270, Life Technologies), 100 units/mL penicillin, and 100 *μ*g/mL streptomycin (PAA Laboratories GmbH, Austria). The cells in each well were washed with PBS and mammalian Ringer solution containing 130 mM NaCl, 4 mM KCl, 1 mM CaCl_2_, 1 mM Mg_2_SO_4_, 1 mM NaH_2_PO_4_, 20 mM HEPES, and 20 mM D-glucose, pH 7.4. The uptake of cGMP was tested after incubation of the cells for 5 min with 100 nM [^3^H]cGMP (PerkinElmer, Hamburg, Germany) and 9.9 *μ*M unlabeled cGMP (BioLog, Bremen, Germany), with and without potential inhibitors at 37°C. Substances investigated for their inhibitory potential were adipic acid (Sigma Aldrich), suberic acid (Sigma Aldrich), glycolic acid (Sigma Aldrich), citric acid (Merck), 3-hydroxyisobutyrate (Fluka),* cis*-aconitic acid (Sigma Aldrich), homovanillic acid (Sigma Aldrich), indomethacin (Sigma Aldrich), sitagliptin (Santa Cruz Biotechnologies), miglitol (Santa Cruz Biotechnologies), captopril (Sigma Aldrich), enalapril (Sigma Aldrich), furosemide (Sigma Aldrich), and bumetanide (Sigma Aldrich). After incubation with radio-labeled cGMP and potential inhibitors, cells were washed three times with PBS at 4°C, and cell lysis was induced by incubation for 2 h with 500 *μ*L of 1 M NaOH. Thereafter, cell lysates were transferred to scintillations vials, 2.5 mL Lumasafe scintillation solution was added to each vial, and radioactivity was counted by a liquid scintillation counter (Tri-Carb 1500; PerkinElmer). Total protein concentrations were determined by the Bradford protein assay, and the cGMP uptake was calculated per milligram of total protein.

### 2.6. Statistical Analysis

Real-time PCR data and data of transport experiments are presented as mean ± SEM. Statistical analysis of real-time PCR data was performed with two-way analysis of variance (ANOVA). Following two-way ANOVA, Bonferroni test was used for multiple comparison of males versus females and of lean versus obese rats (GraphPad Prism 4, version 4.03; GraphPad Software, La Jolla, CA). Data of transport experiments were statistically analyzed with two-tailed unpaired* t*-test (GraphPad Prism 4). Differences were considered significant at the level of *P* < 0.05.

## 3. Results

### 3.1. Structural Changes in the Kidneys of Lean and Obese ZSF1 Rats

**Figure 1:**
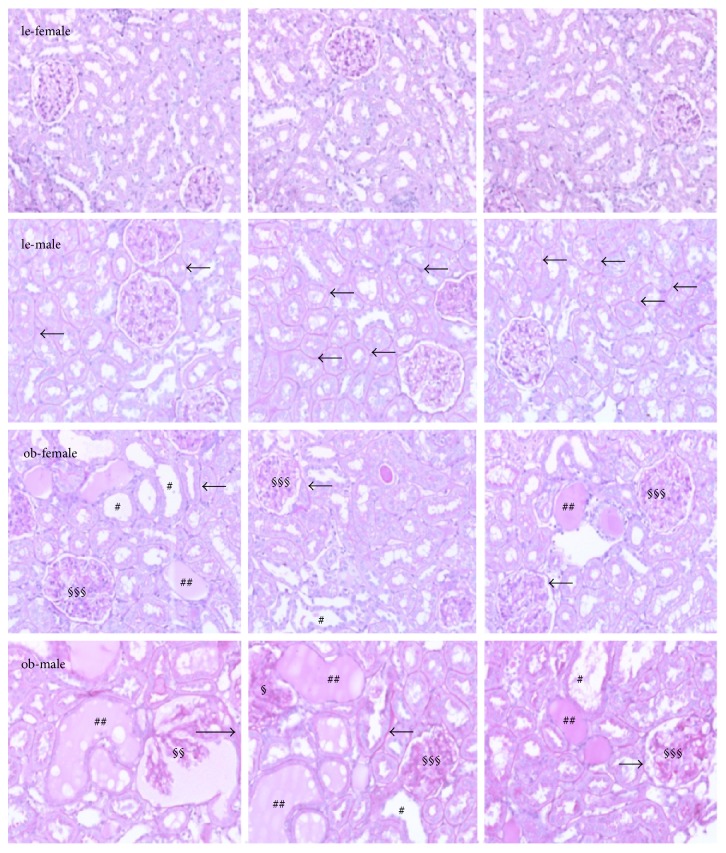
PAS staining in renal cortical slices. Renal sections of lean (le) and obese (ob) ZSF1 rats were stained with PAS and structural changes were analyzed. Examples for histological changes in renal tissue are marked as follows: #, tubular atrophy and dilatation; ##, hyaline protein casts in tubular lumen; §, pressure-induced deformation of glomerulus; §§, dilatation of Bowman's capsule and protein cast in Bowman's space; §§§, glomerulosclerosis. The arrows mark the thickening of tubular and glomerular basement membranes. Magnification: 200x. Representative images from three different rats under each condition are shown.

Light microscopy of periodic acid-Schiff stained sections revealed differences between lean females and lean males in the structure of glomeruli and tubuli of ZSF1 rats (Figure 1, le-female, le-male). Whereas the structure of glomeruli was similar between the two sexes, the tubular basement membrane of lean males appeared to be thicker than that of lean females (arrows). Sex-differences became more obvious in obese rats where renal damage was more prominent in obese males than in obese female ZSF1 rats. In kidneys of obese male and female ZSF1 rats, glomerulosclerosis, extensive mesangial matrix accumulation, and mesangial hypercellularity were detected (Figure 1, §§§). In obese males, in addition, a dilatation of Bowman's capsule and tuft-to-capsule adhesion was present (§§). Tubular injury was indicated by the thickening of tubular basement membrane, tubular dilatation with epithelial cell flattening, tubular lesions, and lumen containing protein as well as by atrophic tubuli (#) with hyaline casts (Figure 1, ##).

Excessive depositions of fibronectin confirmed the development of interstitial fibrosis in kidneys of obese ZSF1 rats. Protein expression of fibronectin was higher in kidneys of lean males than of lean females (data not shown).

### 3.2. Differences between Lean and Obese ZSF1 Rats and Sex-Differences in Renal Cortical mRNA Expression of Transporters and Transcription Factors

The housekeeping genes *β*-actin and cyclophilin A were differently expressed between lean and obese animals and between the sexes (data not shown). In contrast, the expression of Hprt1 did not differ between experimental groups and was, therefore, used as a reference gene in our study.

**Figure 2:**
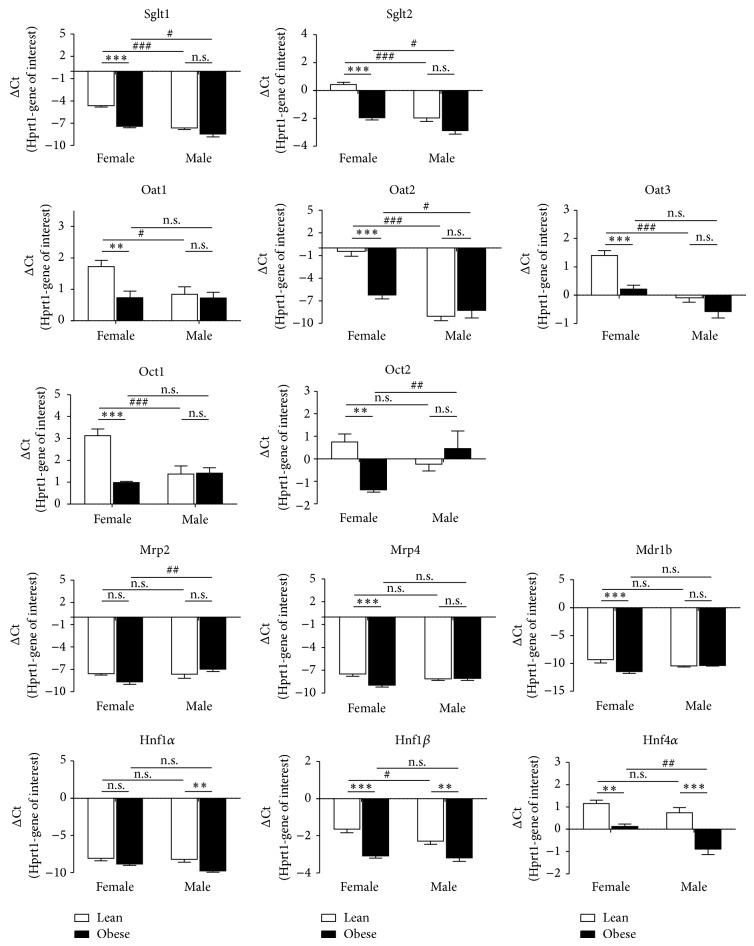
Diabetes- and sex-dependent renal gene expression in lean (le) and obese (ob) ZSF1 rats. Gene expressions were analyzed using TaqMan real-time PCR and presented as mean ± SEM. *n* = 6–8. n.s., not significant; ^**^ *P* < 0.01; and ^***^ *P* < 0.001, for the comparison of ΔCt values between lean and obese ZSF1 rats. ^#^ *P* < 0.05; ^##^ *P* < 0.01; and ^###^ *P* < 0.001, for comparison of ΔCt values between females and males.

Sex dependences as well as differences in mRNA expression between lean and obese rats are presented as ΔCt values in Figure 2 and are summarized as 2^−ΔΔCt^ values in Supplementary Figures 1 and 2, respectively. In Figure 2, white bars correspond to lean and black bars to obese ZSF1 rats, respectively. Negative bars indicate that more PCR cycles were needed to reach the threshold for the gene of interest than for the reference gene Hprt1, that is, the gene of interest shows a lower expression than Hrpt1. Conversely, positive bars in Figure 2 indicate a lower number of PCR cycles for the gene of interest than for Hprt1, that is, a higher gene expression as compared to Hprt1. In general, the more positive (or the less negative) the ΔCt values are, the higher the expression of the gene of interest is.

In lean ZSF1 rats, mRNAs coding Sglt1 and Sglt2 were higher expressed in females, because more PCR cycles were needed to reach the threshold in males (Figure 2). In terms of 2^−ΔΔCt^ values, sex differences amounted to 8.35 ± 1.02-fold for Sglt1, and to 5.51 ± 0.68-fold for Sglt2 (Supplementary Figure  1A). Female expression of Sglt1 and Sglt2 mRNA was retained in obese animals but was less pronounced (Figure 2). Interestingly, Sglt1 and Sglt2 expression was higher in female lean ZSF1 rats than in female obese rats. In males, no significant differences between lean and obese ZSF1 rats were observed for Sglt1 and Sglt2 (Figure 2).

Similarly, levels of Oat1, Oat2, and Oat3 were higher in kidneys of lean females than lean males (Figure 2). These sex-differences vanished for Oat1 and Oat3 in obese rats (Figure 2). In contrast, Oat2 mRNA was higher in obese female than in obese males, with a 6.70 ± 1.58-fold difference (Supplementary Figure  1B). In female obese ZSF1 rats, a decreased expression of Oat1, Oat2, and Oat3 was observed in comparison with lean females (Figure 2). Interestingly, expression of Oat2 showed the strongest difference between lean and obese females. For Oat1, Oat2, and Oat3 mRNA levels no significant differences were detected between male lean and obese ZSF1 rats (Figure 2).

In lean female ZSF1 rats, the mRNA expression level of Oct1 was significantly higher than in their male counterparts and this sex dependence vanished in the kidneys of obese animals (Figure 2). Renal mRNA expressions of Oct1 and Oct2 were significantly reduced in female obese ZSF1 rats as compared to lean females. No significant changes in Oct1 and Oct2 levels were detected between lean and obese male rats. In contrast, significantly higher Oct2 expression was detected in obese males as compared to obese females (Figure 2).

Lean female rats showed a higher Mrp4 and Mdr1b expression than obese females; however, no change in Mrp2 mRNA. In male ZSF1 rats, expression of Mrp2, Mrp4, and Mdr1b remained unchanged in obese rats compared to leans (Figure 2). Renal expression of Mrp2, Mrp4, and Mdr1b showed no significant sex dependence in lean ZSF1 rats. In obese animals, Mrp2 mRNA was higher in males than in females (Figure 2).

The expression of the transcription factor Hnf1*α* was low in all tested animal groups with a detection limit beyond 35 amplification cycles. Hnf1*β* and Hnf4*α* mRNA expressions were significantly reduced in both female and male obese ZSF1 rats as compared to lean controls (Figure 2). The expression of Hnf1*β* was slightly higher in lean females as compared to lean males but was similar in kidneys of obese females and obese males. The transcription factor Hnf4*α* was higher expressed in females as in males only in obese ZSF1 rats (Figure 2).

### 3.3. Inhibition of OAT2 Transport Function

Given the large changes in Oat2 expression, the inhibitory potential of metabolites (adipate, suberate, glycolate, citrate, 3-hydroxyisobutyrate,* cis*-aconitate, and homovanillate) associated with diabetic kidney disease [26, 27], drugs for treatment of diabetes (sitagliptin, miglitol), and hypertension (captopril, enalapril, furosemide, and bumetanide) on human OAT2 expressed in HEK293 cells was investigated.

**Figure 3:**
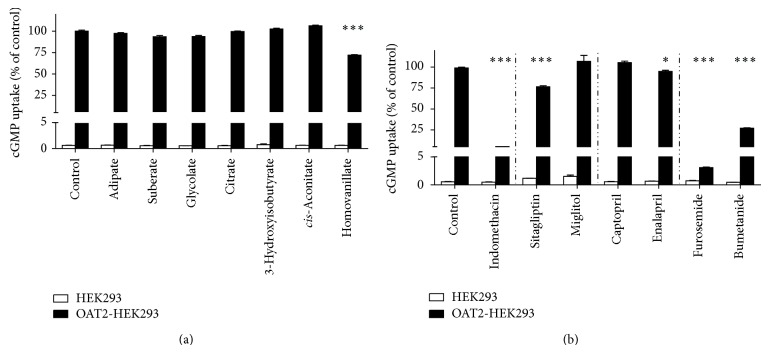
Influence of dicarboxylates, metabolites, and drugs on OAT2-mediated cGMP uptake in HEK293 cells. The uptake of cGMP was determined after 5 min incubation with 10 *μ*M cGMP (0.1 *μ*M [^3^H]cGMP + 9.9 *μ*M unlabeled cGMP) alone (controls) or in the presence of 500 *μ*M potential inhibitors at 37°C. (a) Dicarboxylates and metabolites; (b) drugs. Data are presented as mean ± SEM. *n* = 3. ^*^ *P* < 0.05; ^***^ *P* < 0.001, compared to control.

The OAT2-dependent accumulation of radioactive labeled cGMP, a known substrate for OAT2 [28], was not affected by dicarboxylates adipate and suberate (Figure 3(a)). In addition, cGMP uptake was not inhibited by the metabolites glycolate, citrate, 3-hydroxyisobutyrate, and* cis*-aconitate. In contrast, cGMP uptake was significantly decreased in the presence of the dopamine metabolite homovanillate (Figure 3(a)).

**Figure 4:**
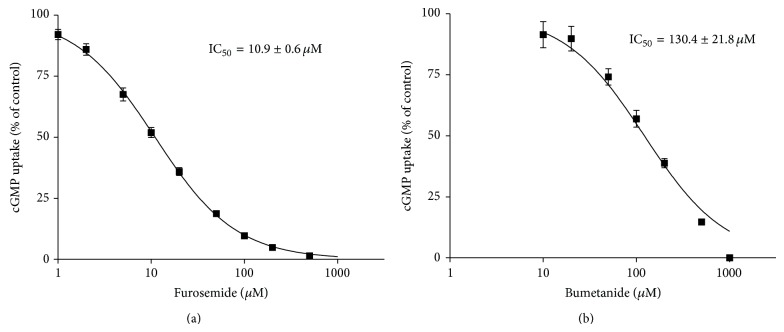
IC_50_ determination for the inhibition of OAT2-mediated cGMP uptake by furosemide and bumetanide. In HEK293 cells stably transfected with OAT2 or empty vector, intracellular cGMP accumulation was determined after coincubation with 10 *μ*M cGMP (0.1 *μ*M [^3^H]cGMP + 9.9 *μ*M unlabeled cGMP) and 1–1000 *μ*M furosemide (a) or 10–1000 *μ*M bumetanide (b), respectively, for 5 min at 37°C. The furosemide and bumetanide concentrations causing half-maximal inhibitory effect (IC_50_) on cGMP accumulation in OAT2 expressing cells were calculated. Data are presented as mean ± SEM. *n*_furosemide_ = 2–4; *n*_bumetanide_ = 2.

OAT2 transport function was abolished by indomethacin, a verified inhibitor of OAT2-mediated cGMP uptake [29] (Figure 3(b)). The antidiabetic drug sitagliptin inhibited OAT2-mediated cGMP accumulation by approx. 25% (Figure 3(b)). Miglitol showed no effect on OAT2-dependent cGMP uptake (Figure 3(b)). No or a small significant inhibition of OAT2-dependent cGMP accumulation was observed in presence of the ACE-inhibitors captopril and enalapril (Figure 3(b)). The uptake of cGMP in OAT2-expressing HEK293 cells was strongly reduced by the diuretics furosemide and bumetanide (Figure 3), showing IC_50_ values of 10.9 ± 0.6 *μ*M (Figure 4(a)) and 130.4 ± 21.8 *μ*M (Figure 4(b)), respectively.

## 4. Discussion

Lean and obese ZSF1 rats are hypertensive, but only obese animals develop type 2 diabetes and diabetic nephropathy, exhibiting symptoms comparable to humans [19]. In this study, we show sex-dependent morphological changes in the renal cortex and in the expression of selected proximal tubular transport proteins and transcription factors in lean and obese ZSF1 rats. In addition, we investigated the impact of several metabolites found in the urine of diabetic patients and of drugs used in the treatment of diabetes and diabetes related diseases on the human organic anion transporter 2 (OAT2).

Our histological results are in line with evidence that the symptoms of diabetic renal disease, for example, renal injury, glomerulosclerosis, interstitial fibrosis, and elevated urine albumin/creatinine ratios, were more pronounced in adult male compared to female diabetic ZSF1 rats [23]. Hypertension, which was reported to be also stronger in male compared to female ZSF1 and spontaneously hypertensive heart failure (SHHF) rats was associated with higher rates of progression of glomerulosclerosis and increased fibronectin expression [30]. Thickening of tubular basement membrane observed in our study was evident not only in kidneys of obese but also in kidneys of lean male ZSF1 rats.

It has already been shown in the kidneys of Wistar rats that Sglt1 and Sglt2 proteins are higher expressed in females than in males [31]. Accordingly, in our study, lean female rats showed considerably higher mRNA levels of Sglt1 and Sglt2 than lean male rats. Recent data suggested that expression of both glucose transporters was increased in obese male Zucker rats at the age of 21 weeks [32]. We found that diabetic nephropathy in obese animals resulted in a decline in the expression of both glucose transporters. The explanation for this discrepancy is possibly the different stage of diabetic nephropathy in the Zucker obese (ZO) rat model, described in published study and in the ZSF1 rat model, used in our experiments. Our histological data confirm the published ZSF1 studies, which showed renal damage, as characteristic for diabetic nephropathy, in obese ZSF1 rats already before 21 weeks of age [19, 23].

In patients with diabetic nephropathy, a significantly downregulated renal OAT1 and OAT3 gene expression and impaired secretion of organic anions were observed [27]. In agreement with these human data, significant downregulation of Oat1 and Oat3 was detected in obese female ZSF1 rats compared to lean females. In contrast to published data [13], higher Oat1 and Oat3 mRNA levels were detected in kidneys of female compared with male ZSF1 rats. The unexpectedly low expression of Oat1 and Oat3 in lean males could be due to structural changes as visualized by the thickening of tubular basement membrane in these animals.

Our results showed for the first time higher expression of Oct1 in lean female compared with male ZSF1 rats. This finding may have some implications on drug evaluation using rats as opposed to humans, because OCT1 is not expressed in renal tubular cells of human kidneys [14]. OCT2/Oct2, well-known for transport of the antidiabetic drug, metformin, was identified at high levels in human and rat kidneys [33]. In this study, obese males, but not lean male ZSF1 rats, showed higher Oct2 expression compared with female counterparts. In accordance with published data [34], Oct2 was decreased by diabetic nephropathy in obese females, but not in obese male ZSF1 rats.

Using another model of diabetes, Nowicki and colleagues showed increased levels of the efflux transporters, Mrp2 and Mrp4, in Western blots from whole kidneys of male rats with streptozotocin-induced type 2 diabetes [34]. We found in renal cortex no changes for Mrp2 and decreased Mrp4 mRNA levels in obese ZSF1 females compared to lean controls. Similarly, Mdr1b expression was decreased by diabetic nephropathy in our female obese rats. No changes were observed between obese and lean male ZSF1 rats. Additionally, no sex-differences were found for Mrp2, Mrp4, and Mdr1b expressions in lean animals, which is in line with already published data [35]. However, we observed higher Mrp2 expression in obese males compared with female ZSF1 rats. The reason for this sex-dependent Mrp2 expression in ZSF1 rat strain remains to be clarified.

The expression of Sglt1, Sglt2, Oct1, Oat1, and Oat3 was shown to be transcriptionally regulated by Hnf1*α* homodimers and Hnf1*α*/*β* heterodimers [36–39]. Low content of Hnf1*α* mRNA was detected in renal cortex of ZSF1 rats. Thus, it appears unlikely that Hnf1*α* plays a dominant role in the transcriptional regulation of transporter mRNAs. The mRNA level of Hnf1*β* was significantly decreased in obese ZSF1 rats compared with their lean counterparts. Furthermore, the promoters of rat Oat1, Oat3, and Oct1 can be activated by Hnf4*α* [40]. The expression of Hnf4*α* was significantly higher in obese females than in obese male ZSF1 rats and was decreased in both sexes in comparison to lean controls. HNF4*α* has been found to be suppressed in kidneys of patients with diabetic nephropathy as well as diabetic Zucker diabetic fatty (ZDF) rats, a model for diabetic nephropathy different from that used in this study [41]. Our data showed a decline in Hnf4*α* expression in male and female obese diabetic ZSF1 rats which is in accordance with published results [41]. The observed sex-dependent Hnf4*α* expression in obese ZSF1 rats was possibly induced by a higher degree of diabetic nephropathy in male animals as compared to females. Interestingly, despite of reduced Hnf4*α* expression in obese male rats, there was no change in expression of renal transporters potentially regulated by Hnf4*α* compared to the lean animals. Given the fact that lean males exhibited a lower transporter expression compared to lean females, we assume that obesity or diabetes induced further reduction in Hnf4*α* was not sufficient to decrease transporter expression even more.

In accordance with our results, higher renal Oat2 expression was found in females than in males [13, 42]. Diabetic nephropathy significantly decreased Oat2 mRNA in renal cortex of obese female ZSF1 rats. Decreased Oat2 mRNA in female animals is probably the consequence of a higher degree of renal damage in kidneys of obese ZSF1 rats. In males, Oat2 mRNA expression was already very low in lean animals and did not decrease further by diabetes. This result is contradictory to previously published data, which showed increased levels of Oat2 protein in whole kidneys of male diabetic Sprague-Dawley rats [34]. It remains to be clarified why Oat2 expression is different in diabetic kidneys of different rat strains.

In human kidneys, OAT2 is located to the basolateral membrane of proximal tubule cells [14] and is, hence, involved in the uptake of metabolites and drugs from the blood into proximal tubule cells. The renal excretion of adipate and suberate is known to be increased in ketotic episodes of diabetes [26], but neither adipate nor suberate affected OAT2, suggesting no significant role of OAT2 in excretion of these anions in diabetes. The concentrations of glycolate, citrate, 3-hydroxyisoburyrate,* cis*-aconitate, and homovanillate were decreased in the urine of patients with diabetic kidney disease [27]. Our experiments excluded an interaction of these metabolites with OAT2, because only homovanillate was able to inhibit cGMP uptake, but this inhibition was very weak.

The weak inhibition of OAT2-mediated cGMP uptake by high concentrations of sitagliptin and enalapril and the absence of any effect of miglitol and captopril on OAT2 function indicate that OAT2 most likely does not contribute to renal excretion of these antidiabetic and antihypertensive drugs. On the other hand, the loop diuretics furosemide and bumetanide inhibited significantly cGMP uptake by OAT2, in our study. The plasma half-life of furosemide was prolonged in patients with renal insufficiency [43]. For example, patients with mild acute kidney injury (AKI) showed a better response to furosemide than patients with severe AKI [44]. In addition, the effect of furosemide on the fractional excretion of potassium in the urine was higher in healthy volunteers compared to patients with stage III chronic kidney disease [45]. Nevertheless, loop diuretics like furosemide are appropriate in chronic kidney disease for reduction of blood pressure [46].

Our data suggest for the first time the involvement of human OAT2 in the secretion of this loop diuretic because of its high affinity (IC_50_ 10.9 *μ*M). In mouse S_2_ cells transfected with human OAT2, an IC_50_ of 603 *μ*M was published for furosemide [47]. The reason for the disagreement between our results and published data for the interaction of furosemide with human OAT2 is unclear but most likely due to the usage of different expression systems, and different radio-labeled substrates (cGMP versus PGF_2*α*_). Bumetanide inhibited OAT2 with moderate affinity (IC_50_ 130 *μ*M), in good agreement with previous data (IC_50_ 77.5 *μ*M) [47]. These two IC_50_ values are much higher than the free plasma concentration of bumetanide [47], indicating that OAT2 does not appreciably contribute to the renal excretion of bumetanide.

## 5. Conclusion

Kidneys obtained from male and female obese ZSF1 rats revealed tissue damage and significant changes in mRNA content of transporters involved in glucose absorption and drug excretion, for example, Sglt1/2, Oat1/2/3, and Oct1/2. These changes are most probably due to diabetes type 2 in obese ZSF1 rats. Discrete signs of damage were found in lean males, resulting from other, nondiabetic pathophysiological changes, most probably the hypertension. A number of proximal tubular transporters showed higher mRNA expression in females compared with male ZSF1 rats. Diabetes in obese animals decreased transporter expression more in female than in male rats. The highest difference was found for Oat2. Additional experiments showed for the first time the possible involvement of human OAT2 in secretion of furosemide, an often prescribed diuretic for patients suffering from diabetes, hypertension, and related kidney diseases. The altered expression of renal transporters may have an impact on sugar and drug excretion in diabetic nephropathy.

## Supplementary Material

Supplementary figure 1. Sex-different expression of renal genes in lean (le) and obese (ob) ZSF1 rats. Gene expressions were analyzed using TaqMan® real-time PCR. DCt = Hprt1-gene of interest and DDCt = δCtle-female – δCtle-male or DDCt = δCtob-female – δCtob-male, *n* = 6–8. a: *P* <0.05, *P* <0.01, and c: *P* <0.001, for the comparison of δCt values for each gene. Values above the horizontal line of unity represent female-dominantly expressed genes, those below the line of unity genes with higher expression in males.
Supplementary figure 2. Renal gene expression in lean (le) and obese (ob) ZSF1 rats. Gene expressions were analyzed using TaqMan® real-time PCR. DCt = Hprt1 - gene of interest and DDCt = δCtle-female - δCtob-female or DDCt = δCtle-male – δCtob-male, *n* =  6–8. a: *P* < 0.05, b: *P* <0.01, and c: *P* <0.001, for the comparison of δCt values for each gene. Values above the horizontal line of unity represent genes with higher expression in lean as compared to obese rats.

## Figures and Tables

**Table 1:** Blood pressure (BP), blood glucose, plasma glyceride, and urine albumin/creatinine ration in young and old male ZSF1 rats.

	Lean ZSF1	Obese ZSF1	Reference
Systol. BP (mmHg) 8 weeks of age		132 ± 19	[21]
Systol. BP (mmHg) 20 weeks of age	156 ± 14	176 ± 23	[21]
Diastol. BP (mmHg) 8 weeks of age		88 ± 8	[21]
Diastol. BP (mmHg) 20 weeks of age	101 ± 9	103 ± 11	[21]

Blood glucose (mg/dL) 8 weeks of age	114 ± 2.8	147 ± 3.9	[19]
Blood glucose (mg/dL) 29 weeks of age	115 ± 11	424 ± 37	[24]

Plasma triglyceride (mg/dL) 8 weeks of age	49 ± 3	483 ± 46	[19]
Plasma triglyceride (mg/dL) 29 weeks of age	194 ± 23	5200 ± 702	[24]

Urine albumin/creatinine ratio 8 weeks of age	0.03 ± 0.001	0.23 ± 0.04	[19]
Urine albumin/creatinine ratio 16 weeks of age	0.048 ± 0.007	1.203 ± 0.118	[23]
