# Supplementary figures and images for: Sex-Differences in Renal Expression of Selected Transporters and Transcription Factors in Lean and Obese Zucker Spontaneously Hypertensive Fatty Rats
Source: J Diabetes Res. 2015 Jan 29;2015:483238. doi: 10.1155/2015/483238 (PMC4325971; doi:10.1155/2015/483238)

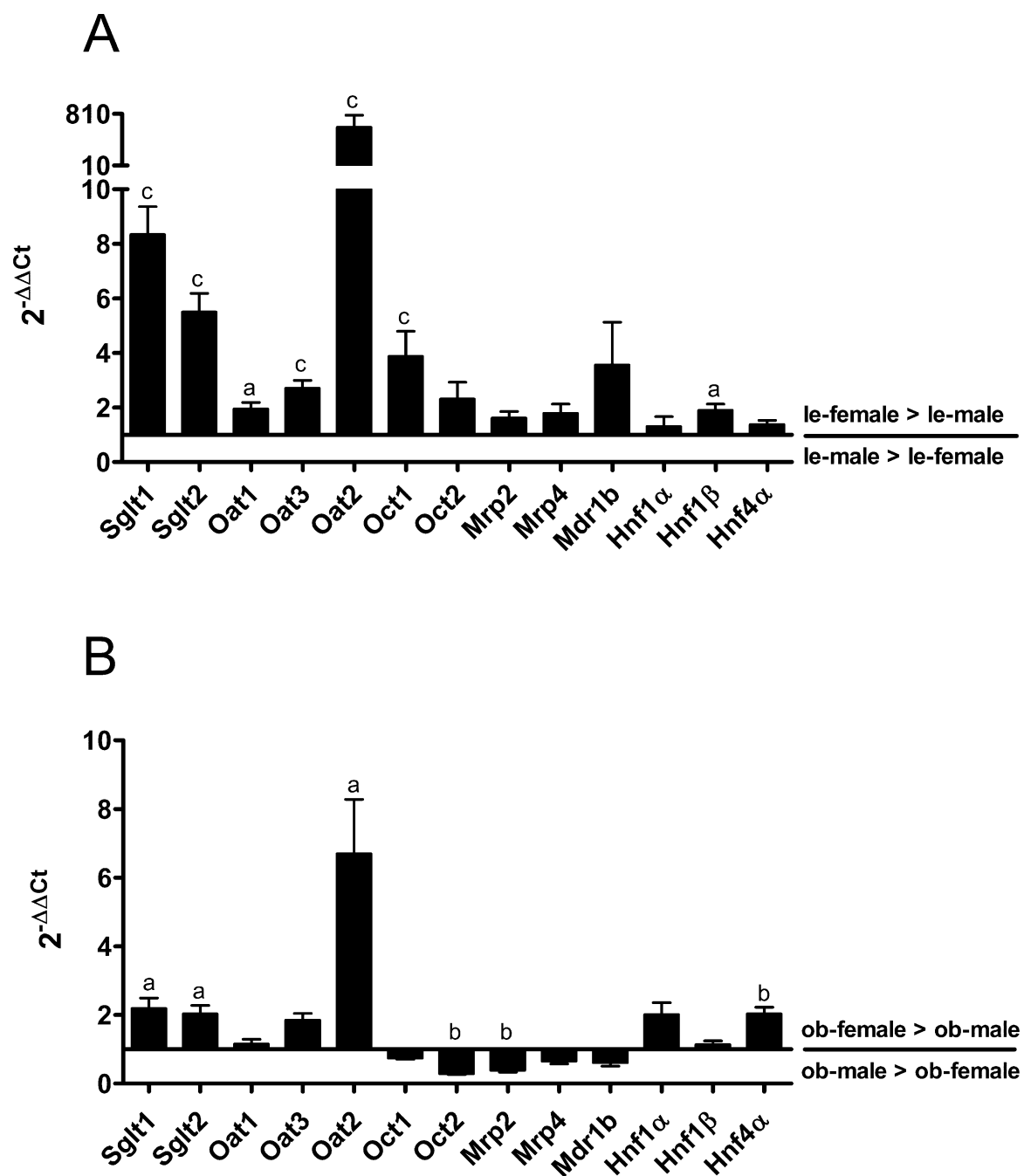

Supplementary figure 1

1

2

3

4

Supplement: Supplementary file 1 — Supplementary figure 1. Sex-different expression of renal genes in lean (le) and obese (ob) ZSF1 rats. Gene expressions were analyzed using TaqMan® real-time PCR. DCt = Hprt1-gene of interest and DDCt = δCtle-female – δCtle-male or DDCt = δCtob-female – δCtob-male, n = 6–8. a: P <0.05, P <0.01, and c: P <0.001, for the comparison of δCt values for each gene. Values above the horizontal line of unity represent female-dominantly expressed genes, those below the line of unity genes with higher expression in males. Supplementary figure 2. Renal gene expression in lean (le) and obese (ob) ZSF1 rats. Gene expressions were analyzed using TaqMan® real-time PCR. DCt = Hprt1 - gene of interest and DDCt = δCtle-female - δCtob-female or DDCt = δCtle-male – δCtob-male, n = 6–8. a: P < 0.05, b: P <0.01, and c: P <0.001, for the comparison of δCt values for each gene. Values above the horizontal line of unity represent genes with higher expression in lean as compared to obese rats. [file 483238.f1.zip › revised Manuscript_Babelova et al 26.pdf]

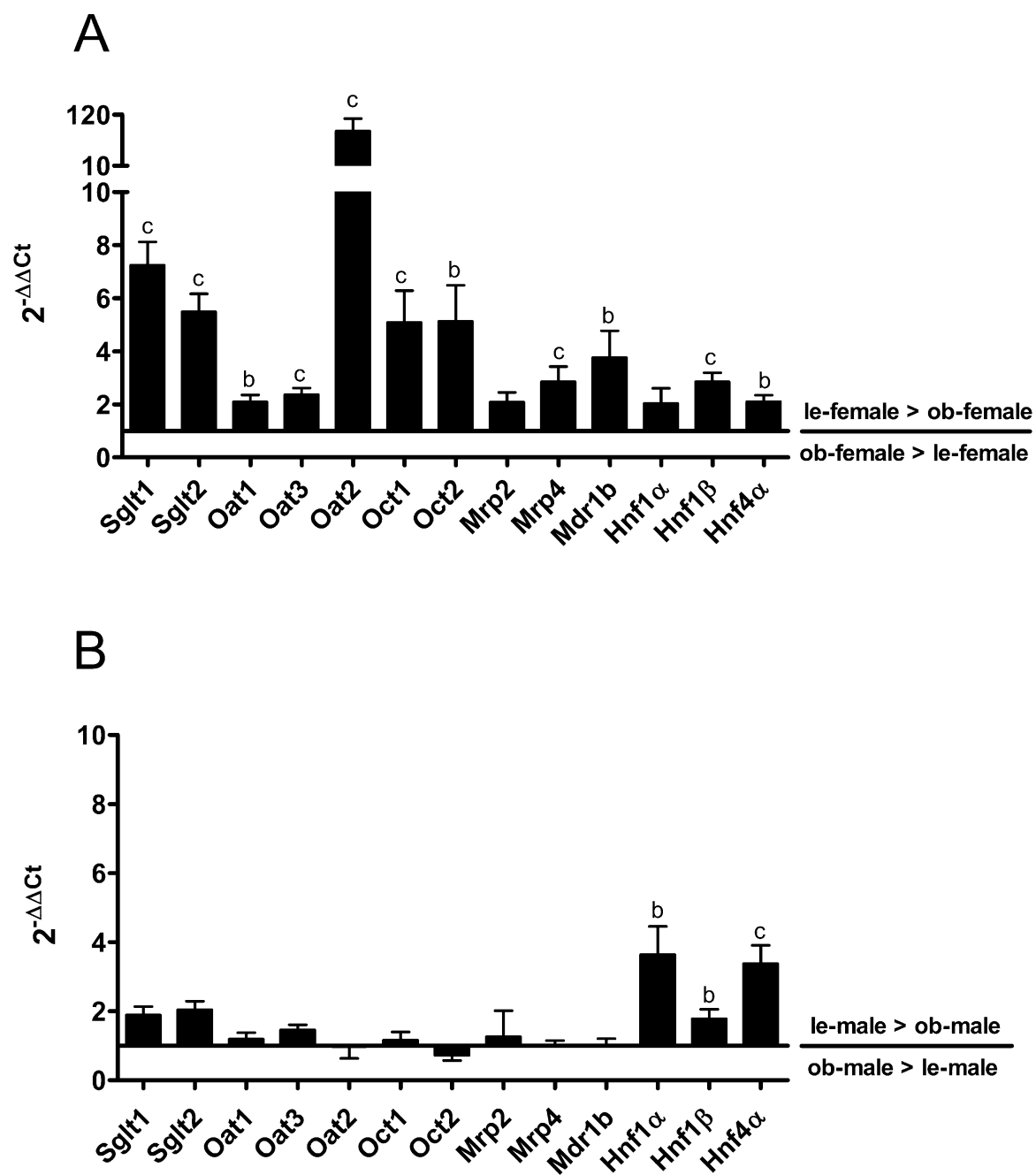

Supplementary figure 2

Supplement: Supplementary file 1 — Supplementary figure 1. Sex-different expression of renal genes in lean (le) and obese (ob) ZSF1 rats. Gene expressions were analyzed using TaqMan® real-time PCR. DCt = Hprt1-gene of interest and DDCt = δCtle-female – δCtle-male or DDCt = δCtob-female – δCtob-male, n = 6–8. a: P <0.05, P <0.01, and c: P <0.001, for the comparison of δCt values for each gene. Values above the horizontal line of unity represent female-dominantly expressed genes, those below the line of unity genes with higher expression in males. Supplementary figure 2. Renal gene expression in lean (le) and obese (ob) ZSF1 rats. Gene expressions were analyzed using TaqMan® real-time PCR. DCt = Hprt1 - gene of interest and DDCt = δCtle-female - δCtob-female or DDCt = δCtle-male – δCtob-male, n = 6–8. a: P < 0.05, b: P <0.01, and c: P <0.001, for the comparison of δCt values for each gene. Values above the horizontal line of unity represent genes with higher expression in lean as compared to obese rats. [file 483238.f1.zip › revised Manuscript_Babelova et al 27.pdf]
